# Extrinsic and intrinsic regulation of DOR/TP53INP2 expression in mice: effects of dietary fat content, tissue type and sex in adipose and muscle tissues

**DOI:** 10.1186/1743-7075-9-86

**Published:** 2012-09-21

**Authors:** Carolin Fromm-Dornieden, Oleksandr Lytovchenko, Silvia von der Heyde, Nina Behnke, Sebastian Hogl, Janina Berghoff, Frederik Köpper, Lennart Opitz, Ulla Renne, Andreas Hoeflich, Tim Beissbarth, Bertram Brenig, Bernhard G Baumgartner

**Affiliations:** 1 Institute of Veterinary Medicine, University of Göttingen, Burckhardtweg 2, 37077, Göttingen, Germany; 2 Statistical Bioinformatics, Department of Medical Statistics, University Medical Center Göttingen, Humboldtallee 32, 37073, Göttingen, Germany; 3 DNA Microarray Facility, Department of Developmental Biochemistry, University of Göttingen, Humboldtallee 23, 37073, Göttingen, Germany; 4 Research Units Genetics & Biometry, Leibniz Institute for Farm Animal Biology (FBN), Wilhelm-Stahl-Allee 2, 18196, Dummerstorf, Germany; 5 Department of Internal Medicine, Metabolic Diseases and Medical Molecular Biology, Paracelsus Private Medical University Salzburg, Müllner Hauptstr. 48, 5020, Salzburg, Austria

**Keywords:** DOR/TP53INP2, High fat diet, Genetically induced obesity, Fat tissue, Muscle tissue

## Abstract

**Background:**

DOR/TP53INP2 acts both at the chromosomal level as a nuclear co-factor e.g. for the thyroid hormone receptor and at the extrachromosomal level as an organizing factor of the autophagosome. In a previous study, DOR was shown to be down-regulated in skeletal muscle of obese diabetic Zucker fa/fa rats.

**Methods:**

To identify sites of differential DOR expression in metabolically active tissues, we measured differences in DOR expression in white adipose tissue (WAT), brown adipose tissue (BAT), skeletal muscle (SM) and heart muscle (HM) by qPCR. To assess whether DOR expression is influenced in the short term by nutritional factors, NMRI mice were fed different fat rich diets (fat diet, FD: 18% or high fat diet, HFD: 80% fat) for one week and DOR expression was compared to NMRI mice fed a control diet (normal diet, ND: 3.3% fat). Additionally, DOR expression was measured in young (45 days old) and adult (100 days old) genetically obese (DU6/DU6i) mice and compared to control (DUKs/DUKsi) animals.

**Results:**

ANOVA results demonstrate a significant influence of diet, tissue type and sex on DOR expression in adipose and muscle tissues of FD and HFD mice. In SM, DOR expression was higher in HFD than in FD male mice. In WAT, DOR expression was increased compared to BAT in male FD and HFD mice. In contrast, expression levels in female mice were higher in BAT for both dietary conditions.

DOR expression levels in all tissues of 100 days old genetically obese animals were mainly influenced by sex. In HM, DOR expression was higher in male than female animals.

**Conclusions:**

DOR expression varies under the influence of dietary fat content, tissue type and sex. We identified target tissues for further studies to analyze the specific function of DOR in obesity. DOR might be part of a defense mechanism against fat storage in high fat diets or obesity.

## Background

Obesity is characterized by an increase of adipose tissue caused by over-nutrition, genetic factors or a combination of both. The two types of adipose tissue in mammals, white adipose tissue (WAT) and brown adipose tissue (BAT) show great differences in cell structure and function.

WAT acts as storage site for fat in the form of triglycerides and it is also known as a key endocrine organ releasing a great number of cytokines, referred to as adipokines, and other molecules that have both local and general effects. About 15% of total body weight consists of WAT in normal weight subjects, which can increase up to 40% in obese humans [1]. BAT, on the other hand, has thermogenic capacity in rodents and humans. BAT is rich in mitochondria and the BAT-specific protein UCP1 (uncoupling protein-1), which accomplishes energy dissipation by uncoupling the respiratory chain from ATP production, thus generating heat (reviewed in [1-4]).

Besides adipose tissue, muscle tissue takes an important part in body composition. Skeletal muscle (SM) composes 35% – 50% of body mass. Significant quantities of plasma fatty acids, either for energy production by fat oxidation or for storage are taken up by SM (reviewed in [5-7]).

Thyroid hormones (THs) regulate metabolism and function of adipose and muscle tissue. They act as pleiotropic factors during development by regulating genes involved in differentiation [1]. Furthermore, THs play an important role in lipid mobilization, lipid degradation, fatty acid oxidation, and glucose metabolism [8]. THs influence the expression of a number of genes involved in lipid and glucose metabolism by direct or indirect effects [8]. In addition, THs stimulate muscle development and differentiation through activation of myogenin and myotube formation in muscle cells [9]. Until the 1980s, THs were used as agents for anti-obesity treatment due to their increasing effect on metabolic rate. However, they show severe side-effects e.g. cardiac acceleration, muscle loss, and other symptoms of thyrotoxicosis. As an alternative, Grover et al. (2007) reviewed the possibility to use the two subgroups of thyroid hormone receptor activators, namely TRα and TRβ [10]. Their specific activation might allow reduction of obesity without the common side-effects of THs [10]. This may be achieved by use of selective molecules or by specific activation of modulators of TH receptors.

DOR (Diabetes and Obesity Related), also called TP53INP2 (Tumor Protein 53 Inducible Nuclear Protein 2), interacts with thyroid hormone receptor α1 (TRα1) enhancing its transcriptional activity. Expression of DOR was shown to be highly reduced in skeletal muscle of obese diabetic fa/fa Zucker rats [9]. DOR knock-down in cultured muscle cells induced to differentiate into mature muscle cells, led to delayed muscle cell differentiation by attenuating expression of myogenin and other TH controlled genes [9]. Effects of DOR on differentiation were also shown in osteoblasts [11]. In liver, DOR is abundantly expressed and in acute-phase reaction it is down-regulated together with other activating factors of the TH signaling system. *TRα2*, which blocks transactivation of TH controlled genes, is up-regulated in these conditions pinpointing the activating role of DOR in the context of the TH system [12]. Mauvezin et al. (2010) demonstrated involvement of mammalian and *Drosophila* DOR in stimulation of the autophagosome formation [13]. Autophagy has been shown to play a role in adipogenesis and fat accumulation [14]. Dysregulation of autophagy may cause impaired insulin sensitivity in obesity [15].

For the analysis of the role of DOR in the pathophysiology of obesity, as an initial step, we determined DOR expression changes in obese mice in comparison to lean animals. For this, DU6 and DUKs mouse strains as well as their inbred lines were used. The mouse strain DU6 was established by selection for high body weight for 70 generations, while the mouse strain DUKs is an unselected, randomly mated control line [16,17].

We hypothesized that DOR might influence adipogenesis as it influences myogenesis having an immediate effect, which requires immediate adaptation of expression. Therefore, we not only used the genetic long-term model, but also selected a short-term animal model using NMRI mice fed different types of fat rich diets to investigate short-term effects of HFD on DOR expression in muscle and fat tissues.

## Methods

### Ethics statement

All procedures were done in accordance with the German Animal Protection Law. Formal approval of the experiment is documented by the approval number “LALLF M-V/TSD/7221.3-1.2-037/06” from the ethical committee of Mecklenburg-Vorpommern under presidency of Dr. Krey.

According to the German law (TierSchG) approval by a named review board was not required.

### Animals and tissues

For the feeding experiment using either a fat diet (FD; 18% fat), a high fat diet (HFD; 80% fat) or a normal fat diet (ND; 3.3% fat), male and female NMRI mice were housed and bred at the mouse facility of the FBN, Dummerstorf, Germany. Mice were housed in a semi-barrier system. Air was exchanged 12 times per hour and coarsely filtered. The room temperature was between 22.4 and 22.7°C, humidity between 50 and 60% and a 12 L:12D light cycle was applied.

**Figure 1:**
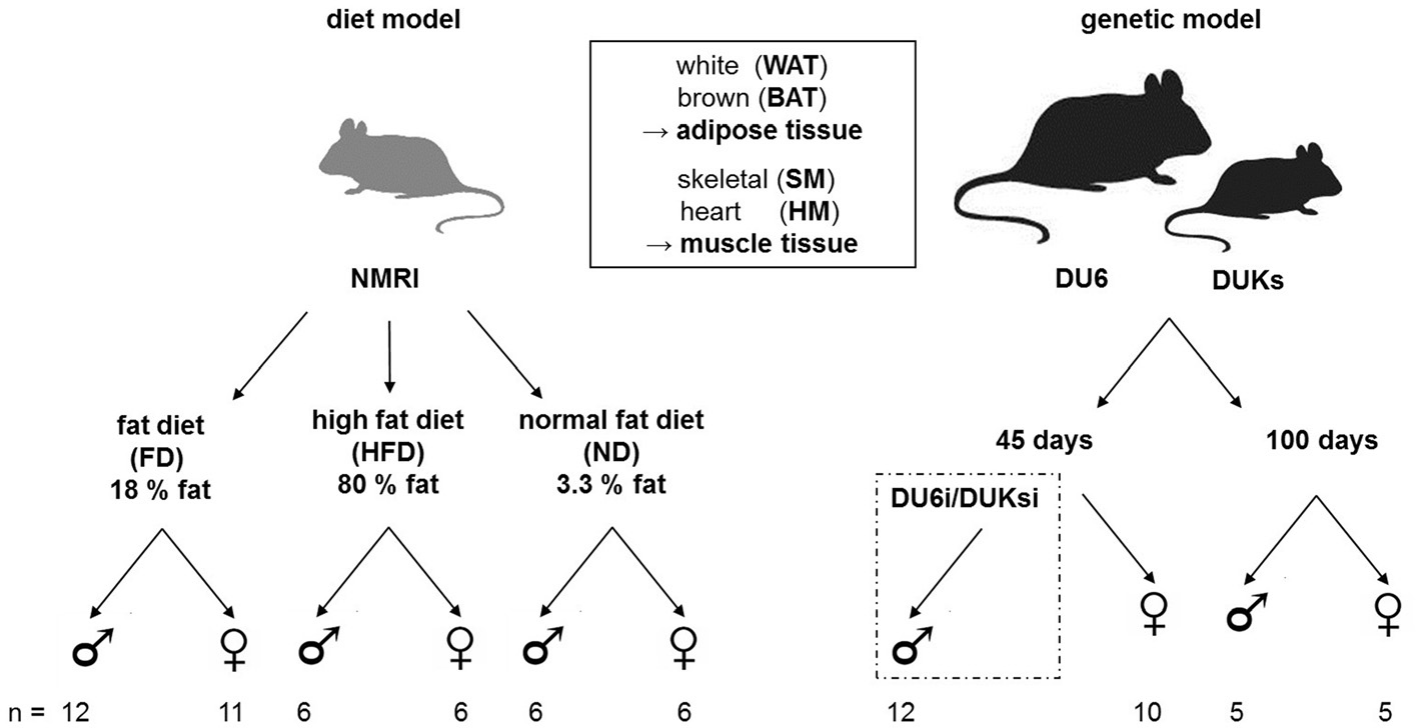
**Overview of investigated mouse models and tissues for DOR expression analysis.** For the diet model, NMRI mice were fed diets with different fat content. In the genetic model, 45 days old male mice from inbred lines DU6i and DUKsi were used (dashed box). All female animals and 100 days old male mice were DU6 and DUKs animals. The maximum number of animals from which tissues were processed is added. Exact counts of animals for each processed tissue are listed in Additional files 3 and 5.

After one week, animals were sacrificed: white (gonadal fat) and brown fat, skeletal muscle tissue (*Musculus quadriceps femoris*) and heart muscle tissue were taken and shock frozen in liquid nitrogen. Before starting the feeding experiments, NMRI mice were fed a standard rodent diet (starch 36.5%, protein 19.0%, fat 3.3% [source: mainly grain and small quantities of soy], raw fiber 4.9%, ash 6.4%, metabolizable energy: 12.8 MJ/kg; Ssniff, Soest, Germany; catalogue number V1534-0) and water *ad libitum* until the age of 40 days. The FD (polysaccharides 30.4%, disaccharides 11.1%, protein 17.6%, fat 18.0% [source: sun flower oil], raw fiber 3.8%, ash 5.9%, metabolizable energy: 17.2 MJ/kg; Altromin, Lage, Germany; catalogue number C1057) and the HDF (starch 0.6%, protein 8.0%, fat 79.2% [source: lard], raw fiber 5.0%, ash 4.5%, metabolizable energy: 28.6 MJ/kg; Ssniff, Soest, Germany; catalogue number E15149-30) were administered to experimental animals for one week *ad libitum*. Fat composition between standard diet and FD was highly similar containing high amounts of unsaturated fatty acids, while HFD contained mainly saturated fatty acids. Age matched animals on normal fat diet ND were used as control (animals used are described in Table 1, the study design is outlined in Figure 1). The mouse strain DU6 was established by selection for high body weight. The mouse strain DUKs is an unselected, randomly mated control line [17]. Lines DU6i and DUKsi are inbred lines split from DU6 and DUKs in generation 79 and then full-sib mated for 41 generations. DU6/DU6i and DUKs/DUKsi lines were bred at the mouse facility of the FBN, Dummerstorf, Germany (see above and reviewed in [16,17]). Fixed formula food for laboratory mice (Altromin R 1314: protein 22.5%, fat 5%, raw fiber 4.5%, ash 6.5%, metabolizable energy: 12.5 MJ/kg; Altromin GmbH, Lage, Germany) was supplied *ad libitum* and fresh tap water was provided. For tissue isolation, male and female animals were sacrificed at day 45 *post natum* (*p.n*.; male DU6i/DUKsi and female DU6/DUKs mice) and day 100 *p.n.* (male and female DU6/DUKs mice), tissue samples were obtained as described before (Figure 1). From DUKsi male mice at day 45 additionally liver and pancreas were taken. 

**Table 1:** Mouse models used in this study (values are expressed as mean +/−SD; “-” indicates that data are not available)

**mouse line**	**model**	**special treatment**	**from generation (m/f)**	**age (days) at section**	**sex (n)**	**mean BW at section (g)**
NMRI		fat diet FD: 18% fat		48	male (12)	34.27
						+/− 2.24^*^
					female (11)	25.22
						+/− 1.54^*^
		high fat diet HDF: 80% fat		48	male (6)	-
					female (6)	-
		normal fat diet ND: 3.3% fat		48	male (6)	33.87
						+/− 1.47
					female (6)						27.38
						+/− 2.41					
DU6	selection line: obese line		137 (0/10)	45	female (10)	64.40					
						+/− 3.24					
			133 (5/5)	100	male (15)	104.95					
						+/− 14.67					
			137 (10/10)	100	female (15)	82.17					
						+/− 8.57					
DUKs	control line: normal line		137 (0/10)	45	female (10)	25.03					
						+/− 2.69					
			133 (5/5)	100	male (15)	37.41					
						+/− 2.57					
			137 (10/10)	100	female (15)	35.81					
						+/− 4.85					
DU6i	selection line: obese line	inbreeding for the last 47 generations	126 (12)	45	male (12)	63.18					
						+/− 5.46					
DUKsi	control line: normal line	inbreeding for the last 47 generations	126 (12)	45	male (12)	28.98					
						+/− 1.93					

* aberrantly, data were obtained from 6 animals each.

Samples of tissues described above were stored at −80°C and used for RNA isolation, as described below.

### RNA isolation and reverse transcription

For RNA isolation from white (gonadal fat) and brown fat, RNeasy® Lipid Tissue Mini Kit (Qiagen, Hilden, Germany) was used. Tissue pieces of 30–50 mg were cut, immediately placed into 1 ml of QIAzol Lysis reagent, homogenized for 20 s using a rotor-stator homogenizer and further treated according to the manufacturer’s protocol. RNA from *Musculus quadriceps femoris* and heart was extracted with RNeasy® Fibrous Tissue Mini Kit (Qiagen, Hilden, Germany) according to manufacturer’s instructions. DNase treatment for all sample types was performed using RNase-Free DNase Set (Qiagen, Hilden, Germany).

RNA was quantified by spectrophotometry and used for reverse transcription (500 ng RNA/reaction). Reverse transcription was performed using Omniscript® Reverse Transcription Kit (Qiagen, Hilden, Germany) using a poly-dT primer, according to the manufacturer’s protocol.

### Quantitative real-time PCR (qPCR)

Gene expression was quantified by qPCR using QuantiTect® SYBR® Green PCR Kit (Qiagen, Hilden, Germany) according to the manufacturer’s protocol in an MX4000 light cycler (Stratagene Inc, La Jolla, Canada). 1 μl of cDNA was used per reaction; reactions were performed in duplicates. The cycling program consisted of an initial pre-heating step at 95°C for 15 min, followed by 40 three-step cycles: 15 s at 95°C, 30 s at 58°C and 30 s at 72°C. Specificity of the reaction was assessed by melting curves from 55°C to 95°C. No-template controls were included to all reactions to confirm absence of contaminations.

Primers used for qPCR, their annealing temperatures (T°an) and amplification efficiencies are listed in Additional file 1. Primers for mouse housekeeping genes were purchased from RealTimePrimers (RealTimePrimers.com, Elkins Park, PA, USA). Amplification efficiencies (E) for each primer pair were estimated using formula: E=10^−1/slope−1 , where slope of standard curve built on serial 10-fold dilutions was used [18,19].

The data were analyzed using efficiency-corrected ΔΔCt method of relative quantification (see [20,21] and MX4000 instructions, Stratagene).

To minimize mistakes caused by improper normalization, expression of genes of interest was normalized against the geometric mean of multiple normalizers (Additional file 1) using recommendations of Vandesompele et al. (2002) and GeNorm software, cited in there [20]. The primers used for NMRI mice were ACTb, B2M and PPIA, for the genetic models we used ACTb, GUSb and PPIA for normalization.

### Statistical analysis

All expression data from qPCR of metabolically challenged mice were normalized with housekeeping genes and calibrated to reference mice. Normalized data were analyzed using Welch Two Sample t-tests for independent samples. We inspected whether the null hypothesis that the true difference in group means, i.e. animal group of interest vs. related control group, equals zero can be rejected at the significance level of 5%. The tests were conducted in a two-sided fashion but in case of significance also for the specific alternative hypotheses, i.e. true difference in group means being greater than zero or less, respectively. The differences were considered significant in case of p < 0.05, highly significant if p < 0.01 and highest significant if p < 0.001. The t-tests and graphic constructions were performed using the software R [22].

ANOVA was performed with relative data to means of gender, age, obesity model and tissue type matched control animals, i.e. raised under normal dietary condition and DUKs(i) strains, respectively, to prove influences of diet, tissue type and sex on DOR expression levels in different mouse models [23].

## Results

### DOR expression in different tissue types

**Figure 2:**
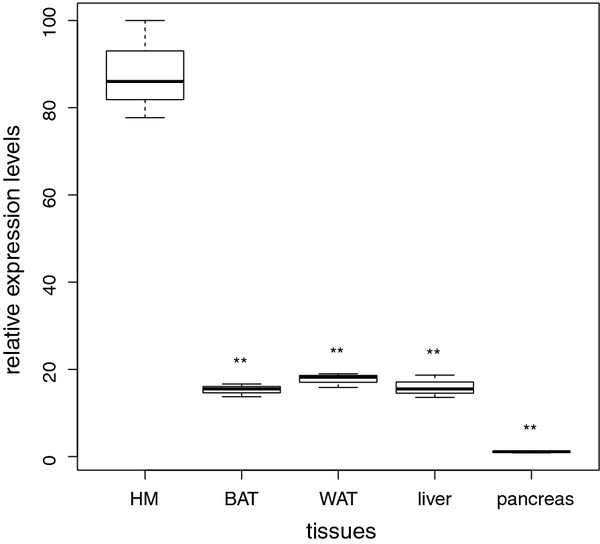
**DOR expression levels in different tissues.** DOR expression levels were measured in different tissues of twelve 45 days old lean male DUKsi mice by qPCR. Expression levels of brown adipose tissue (BAT), white adipose tissue (WAT), liver tissue and pancreas tissue are presented as a percentage of the DOR expression in heart muscle (HM; **p < 0.01). The maximum of expression in HM corresponds to 100%.

DOR is highly transcribed in some insulin-sensitive tissues like skeletal and heart muscle, while expression is lower in others like WAT [9]. In pools from RNA of twelve 45 days old lean male DUKsi mice, DOR expression levels of heart muscle (HM), brown adipose tissue (BAT), white adipose tissue (WAT), liver tissue and pancreas tissue were measured by qPCR. We reconfirmed that DOR transcription is highest in heart muscle (Figure 2). Expression of DOR in WAT, BAT and liver is significantly lower (p < 0.01) and equates approximately 20% to the expression in heart muscle. Additionally, DOR expression in pancreas corresponds approximately to 1.5% of the DOR expression in heart muscle (p < 0.01). 

### DOR expression in models of obesity

DOR expression and its changes in different tissues in models of obesity were investigated in two systems: in FD and HFD mice and in young and adult mice in a genetic system (Table 1 and Figure 1).

### DOR expression in FD/HFD mice

In order to investigate changes in DOR expression caused by high fat diet, 40 days old NMRI mice were fed two different fat diets (FD: 18% fat or HFD: 80% fat) for one week. After one week of feeding, weights of the animals did not differ significantly from starting values (Table 2). It is noteworthy that food consumption in FD was generally lower than in ND, with females gaining less weight in FD, which is in accordance to other studies [24]. Caloric intake in males was identical between ND and FD feeding, while female caloric intake declined in FD (Table 2). We calculated the average food intake by division of the total food intake by the average weight during the feeding phase. Average calorie intake has been calculated accordingly. Female animals consumed slightly more food and ingested slightly more calories per gram body weight than male animals in both groups, control diet and fat diet (Table 2). Weight data for HFD animals are not available. 

**Table 2:** Overview of changes in body weight and food consumption in NMRI mice fed a normal diet (ND; 3.3% fat) or fat rich diet (FD; 18% fat) for one week (values are expressed as mean +/−SD)

**diet**	**sex (n)**	**mean BW (g)**		**changes in BW (g)**	**FC (g)**	**FC (kJ)**	**FC (g)/mBW**	**FC (kJ)/mBW**
		**day 1**	**day 8**					
FD 18% fat	male (6)	32.02	34.27	+ 2.25	35.47	610.03	1.07	18.40
		+/− 2.47	+/− 2.24	+/− 0.62	+/− 2.71	+/− 46.63		
FD 18% fat	female (6)	25.62	25.22	- 0.40	28.28	486.47	1.11	19.14
		+/− 1.51	+/− 1.54	+/− 1.98	+/− 3.54	+/− 60.96		
ND 3.3% fat	male (6)	32.92	33.87	+ 0.95	48.07	615.25	1.44	18.42
		+/− 1.30	+/− 1.47	+/− 0.79	+/− 6.20	+/− 79.40		
ND 3.3% fat	female (6)	25.58	27.38	+ 1.80	41.98	537.39	1.59	20.29
		+/− 1.46	+/− 2.41	+/− 1.24	+/− 6.52	+/− 83.46		

Changes in body weight (BW), food consumption (FC) and food consumption expressed as kilo Joule (FC (kJ)) in NMRI mice fed a normal diet (ND; 3.3% fat) or fat rich diet (FD; 18% fat) for one week; average food intake per gram body weight was calculated as FC(g)/mean body weight (mBW); average calorie intake per gram body weight was calculated as FC(kJ)/mBW.

At the age of 48 days, DOR expression was assessed by qPCR in white adipose tissue (WAT) and brown adipose tissue (BAT). Additionally, expression levels were measured in skeletal muscle (SM) and heart muscle (HM) (Additional file 2). DOR expression levels were compared to expression levels in the respective tissues of control animals (Additional file 3). After feeding the animals with 18% fat (FD) *ad libitum* for one week, in WAT statistically significant up-regulation of DOR was detected in males (n = 7; p < 0.05). Similar changes were observed in HM of females (n = 11; p < 0.05).

Feeding an 80% fat diet (HFD) led to more prominent changes in DOR expression in WAT of HFD females, with a significant down-regulation of expression (n = 6; p < 0.01). HFD diet led to a significant increase of DOR expression in HM of males (n = 6; p < 0.05). In BAT and SM of male and female mice, changes in DOR expression were not statistically significant (Additional file 3).

**Figure 3:**
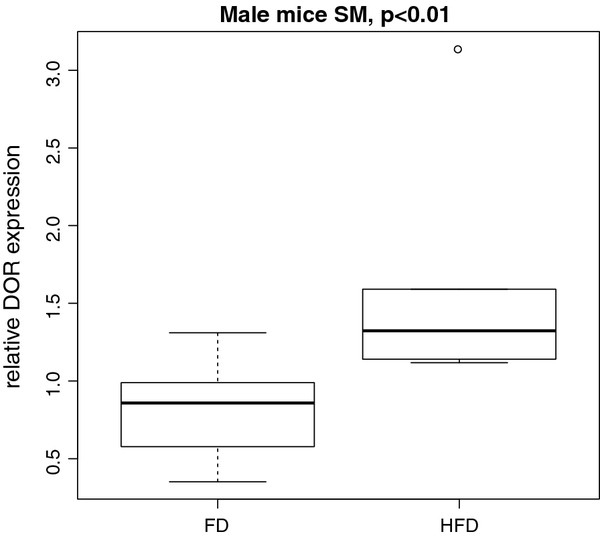
**Influence of diet on DOR expression in SM of male mice.** Influence of different fat rich diets (FD = 18% fat content; HFD = 80% fat content) over one week *ad libitum* on DOR expression was proved by ANOVA. We found significant influence of diet in SM of male mice in FD and HFD animals (normalized to control animals). Changes in DOR expression in comparison to control animals were more prominent in HFD mice.

Influences of diet, tissue type and sex on DOR expression were tested by ANOVA. For diet, there was a general effect on DOR expression detected for WAT and BAT (p < 0.05) (Table 3). Additionally, diet has a significant influence on DOR expression in SM of male mice (p < 0.01) (Figure 3). The expression levels of DOR were higher in HFD- than in FD-fed animals.

**Table 3:** General effects of diet, tissue weight and sex in metabolically challenged mice (ANOVA analysis; “NS” indicates that effects are not significant)

	**adipose tissue (BAT/WAT)**		**muscle tissue (SM/HM)**	
	**factor**	**p-value**	**factor**	**p-value**
FD/HFD mice	diet	0.024470	diet	NS
	tissue	NS	tissue	0.00372
	sex	0.007940	sex	NS
genetically obese mice (100 days old)	tissue	NS	tissue	NS
	sex	0.0438	sex	0.02

BAT, brown adipose tissue; WAT, white adipose tissue; SM, skeletal muscle; HM, heart muscle; FD, 18% fat diet; HFD, 80% fat diet.

**Figure 4:**
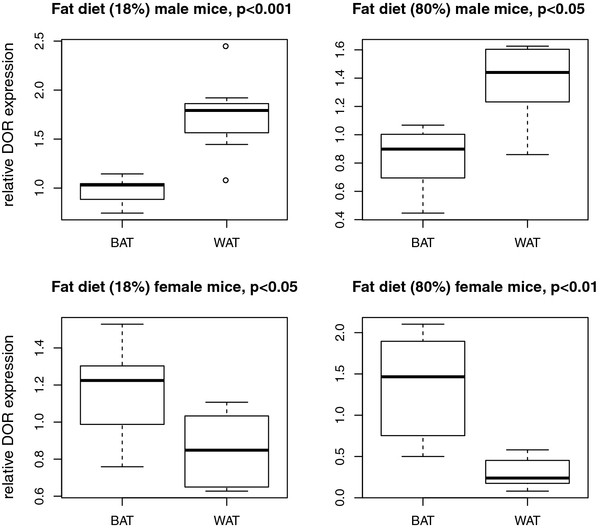
**Influence of tissue type on DOR expression in mice fed a high fat diet.** Influence of adipose tissue type (BAT and WAT) on DOR expression was proved by ANOVA. We found significant influence of tissue type in male and female mice in FD and HFD animals (normalized to control animals). In male mice DOR expression was higher in WAT while in female animals the expression levels were higher in BAT.

In FD and HFD animals, tissue type also influenced DOR expression (Figure 4). DOR mRNA levels were higher in WAT than in BAT of male animals (p < 0.05). In female mice, DOR expression was higher in BAT (p < 0.05). Additionally, a general effect was detected for HM and SM (p < 0.01) (Table 3). In male FD mice, DOR expression was higher in HM than SM (p < 0.05) (Additional file 4).

**Figure 5:**
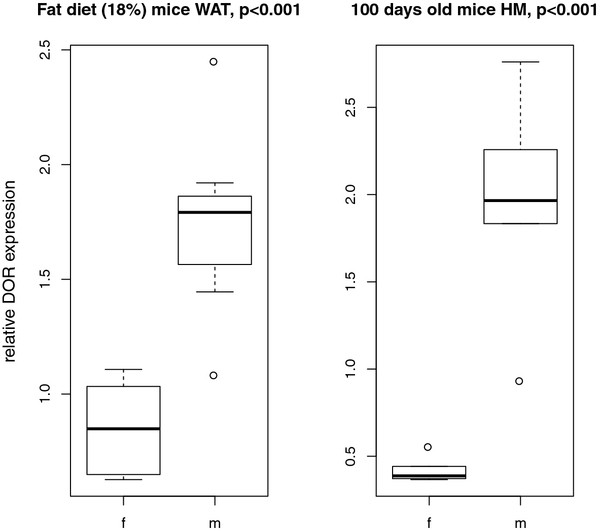
**Influence of sex on DOR expression in WAT and HM of metabolically challenged mice.** Influence of sex on DOR expression in different tissues was proved by ANOVA. We found significant influence of sex in WAT of FD mice and in HM of 100 days old DU6 animals (normalized to control animals). In both cases, DOR expression was higher in male than female mice.

An influence of sex on DOR expression was detected for BAT and WAT (p < 0.01), which was stronger than the influence of diet. In WAT of FD animals, DOR expression was significantly higher in male mice than in female ones (p < 0.001) (Figure 5).

### DOR expression in DU6/DU6i and DUKs/DUKsi mice

Four mouse lines were used for this part of the study. Two of them, long-term selected for increased body weight (DU6/DU6i), served as models for genetically predisposed obesity. The other strains (DUKs/DUKsi) were bred as normal weight controls (see [16,17], Figure 1 and Table 1).

DOR expression was determined in WAT, BAT, SM and HM in male and female animals at day 45 *p.n.* and day 100 *p.n.*, corresponding to juvenile (day 45 *p.n.*) and adolescent (day 100 *p.n.*) life phases. Expression levels in DU6/DU6i were compared to DUKs/DUKsi mice in tissues of age and sex matched groups (Additional files 5 and 6).

In 45 days old male animals, DOR mRNA levels were significantly lower in HM of obese (DU6i) mice compared to normal weight (DUKsi) ones (n = 12; p < 0.05). Conversely, in 100 days old animals, DOR was expressed significantly higher in HM of male obese DU6 mice (n = 5; p < 0.05) (see Additional file 5).

In BAT, DOR expression was higher in obese 100 days old female DU6 animals compared to normal weight DUKs ones (n = 5; p < 0.05). In WAT and SM of male and female DU6/DU6i mice, no statistically significant difference in expression was found.

Influences of sex and tissue type on DOR expression were tested by ANOVA. A general influence of sex on DOR expression was detected for all tissues (p < 0.05) (Table 3). In HM of 100 days old animals, DOR expression was significantly higher in male mice than female ones (p < 0.001), (Figure 5).

In genetically obese animals, tissue type influenced DOR expression only in WAT and BAT of 45 days old male mice (Additional file 7). DOR expression was higher in WAT than in BAT (p < 0.01).

## Discussion

Based on the fact that DOR is drastically down-regulated in SM of obese diabetic Zucker fa/fa rats and modulates expression of TH controlled genes [9], we investigated DOR expression in FD/HFD fed mice and genetically obese mice in different tissues. Differences in DOR expression were measured in diverse types of adipose and muscle tissue by qPCR. To test short-term influences on DOR expression by nutritional factors, mice were fed different fat rich diets with 18% or 80% fat content for one week and DOR expression was compared to normal fed animals (Figure 1, Table 1). In SM, DOR expression was higher in HFD than in FD male mice (Figure 3). In WAT, DOR expression was increased compared to BAT in male FD and HFD mice. In contrast, expression levels in female mice were higher in BAT for both dietary conditions (Figure 4).

Additionally, long-term effects on DOR expression were investigated in genetically obese mice compared to control animals. For this, tissues were derived from young (45 days old) and adult (100 days old) animals (Figure 1, Table 1). DOR expression levels in all tissues of 100 days old genetically obese animals were mainly influenced by sex. In HM, DOR expression was higher in male than female animals (Figure 5).

Here we show that DOR expression varies under the influence of dietary fat content, tissue type and sex.

### Influence of dietary fat content on DOR expression

High fat diets are known to induce significant changes in the muscle and adipocyte transcriptomes. Even short-term feeding with a diet containing 45% calories in a form of fat alters expression of more than 1000 genes in skeletal muscle, with a fold change >1.3; most of these genes are primarily involved in tissue development and morphogenesis [25]. Long-term (4–6 weeks) feeding with high fat diets also leads to changes in transcription of hundreds of genes, mostly related to immune response and energy metabolism, both in adipocytes and muscle cells [25-27].

In this study, we measured DOR expression after one week of FD or HFD and notably DOR expression levels already displayed significant changes in WAT and HM. Via ANOVA we tested whether the type of diet, i.e. FD and HFD, significantly influences DOR expression in skeletal muscle tissue of male mice. Expression values were relative to control mice under ND as described in “Statistical analysis”. We observed that HFD increased DOR expression significantly stronger (p < 0.01) compared to the FD as displayed in Figure 3. In WAT and BAT, we demonstrated an effect of diet in male and female mice (Figure 4). This might indicate that DOR plays a role in fighting negative effects of lipotoxicity or high fat diets in general, influencing thyroid hormone action or modulating autophagy. The description of increased TH serum levels in rats fed a HFD promotes this theory [24]. Additionally, higher thyroid hormone serum levels in obese than in lean women have been described [28].

DOR expression has been compared between the stromavascular fraction (SVF) and mature adipocytes in WAT and VAT [29]. In both tissues, expression of DOR is significantly higher in adipocytes than in the SVF (GSD2818). It cannot be ruled out that DOR expression is changed in SVF influencing the growth of fat pads. This hypothesis is supported by the fact that mutations in *TRα1*, the predominant TH receptor type in adipocytes, lead to changes in the size of different fat pads [30,31].

In rats fed a HFD for 8 weeks, circulating T3 and T4 levels were not elevated [24]. The activity of deiodinase D1, on the other hand, was increased slightly, while activity of D2, which converts T4 into the biologically much more active T3, was either similar to the control group or showed reduced activity in BAT. However, increased serum thyroid stimulating hormone (TSH) levels were associated with up-regulation of thyroid function as observed by increased iodide uptake and D1 activity. A shift toward fat oxidation was observed, which depended mainly on the nutrient composition of the diet.

Rather than changed T4 or T3 levels in serum, transport, processing and modulation of signaling seem to be the driving forces behind the effects of TH. Modulation of TH signaling can be exerted by co-activators/co-repressors or proteins competing with T3 for binding sites on responsive promoters. *PPARγ* was shown to be down-regulated within hours following TH administration [32]. Since PPAR and TR proteins bind to similar DNA sequences, depletion of *PPARγ* might facilitate binding of TR to these sites [33]. As DOR interacts with TRα1 competing with the preferred bonding partner RXR, it also might modulate genomic response to T3 signaling in the early phase of HFD *in vivo*.

The type of fat ingested has been shown to have an impact on health and gene expression and we analyzed data on DOR expression scanning data in “GEO profiles”. In rats fed diets with same caloric values but different fat compositions [34], DOR expression levels were increased in liver of lard diet fed animals, but not in others such as olive oil, coconut oil or cod liver oil diet (GDS1307). In a human study, abdominally overweight patients were subjected to two different diets: one rich in saturated fatty acids (SFA) and one rich in mono-unsaturated fatty acids (MUFA) for 8 weeks [35]. No difference in DOR expression was detected, neither in the SFA nor in the MUFA group. Analysis by sex, however, revealed a positive effect of SFA diet in women, but not in men (GDS3678), again confirming the importance of sex on the expression of DOR (see “Influence of sex on DOR expression”). In a setting to identify genes that promote weight gain [27], DOR mRNA levels were found to be significantly higher in the epididymal fat of high weight gainers than in low weight gainers following ingestion of a high fat diet (GDS2319).

Fasting, on the other hand, seems to influence DOR expression in an opposite manner to a fat-rich diet. After a 24 h fasting period, DOR levels decreased significantly in WAT, but not in BAT (GDS3135) in rats [36]. Similar results come from patients who underwent gastric bypass that reduced body-weight by approximately 45% [37]. DOR levels in SM were significantly decreased in SM of treated women (GDS2089).

### Influence of tissue type on DOR expression

In FD (18% fat diet) and HFD (80% fat diet) animals we detected a significant influence of tissue type on DOR expression (Figure 4). In male animals, DOR expression was higher in WAT than in BAT, while in female mice DOR expression was higher in BAT than in WAT.

Thyroid hormone is an important factor for cold-induced thermogenesis [38] and TH supports the effects of norepinephrine on UCP1 expression and brown adipocyte recruitment [39]. Furthermore, T3 has a positive effect on mitochondrial biogenesis [40]. On the other hand, it was shown that HFD does not alter circulating T3 levels but rather the local availability of T3, as discussed above. Up-regulation of DOR in adipose tissue might contribute to increased mitochondrial biogenesis and energy expenditure by increasing the effects of local T3.

Comparing metabolically challenged mice to reference animals, it is a very interesting finding that DOR is up-regulated in male and female heart muscle when fed a FD or HFD (Additional file 3). In the genetic model, DOR expression is down-regulated in the heart muscle of DU6i males at day 45 *p.n.*, up-regulated in DU6 males at day 100 *p.n.* and not altered in females (Additional file 5). TH plays an important role in cardiac mitochondrial biogenesis thus increasing myocardial mitochondrial mass, respiration, OXPHOS enzyme activity, protein synthesis and others (summarized in [41]). In the heart, TRα is the predominant form. Hyperthyroidism leads to increased metabolic rate and increased mitochondrial biogenesis by up-regulation of nuclear genes encoding mitochondrial proteins [42].

DOR up-regulation in FD and HFD animals might be part of an early defense system against effects of lipotoxicity by increasing mitochondrial activity and metabolic rate.

In the FD and HFD, as well as the genetic mouse model, no expression changes were detected in SM, neither in male nor in female animals (Additional file 3 and 5). DOR, however, was shown to be down-regulated in SM of obese type 2 diabetes fa/fa rats, before [9]. We propose that physiological changes conferred by diabetes rather than the obese condition lead to down-regulation of DOR. This needs confirmation by measuring DOR in diabetic animals.

### Influence of sex on DOR expression

In this study, we discovered effects of sex on DOR expression in fat tissue of the diet model and in all tissues of the genetic model. In the diet model, the effect of sex was stronger than the effect of diet (Table 3). Male FD mice showed significantly higher DOR expression in WAT than female animals (Figure 5). In the genetic model we found a similar difference in 100 days old obese mice in HM (Figure 5).

Nearly all common diseases exhibit some degree of sex bias, often being very dramatic [43]. The distribution of fat displays a sexual polymorphism with females depositing relatively more fat in subcutaneous/inguinal depots whereas males deposit more fat in the intra abdominal (summarized in [44]). Also properties of adipocytes differ between fat depot and sex. In a study by Macotela et al. (2009), male GAT adipocytes were 60% larger than those from females [45]. These findings indicate that gene expression needs to be fine-tuned between diverse fat depots in male and female individuals. There are several studies confirming this hypothesis. Yang et al. (2006) compared gene expression in various tissues in 334 mice. They detected thousands of genes to be sexually dimorphic in muscle and fat tissue [43]. These genes exhibited highly tissue-specific patterns of expression and were enriched for distinct pathways. Sexual dimorphisms were also detected for gene expression in C57/Bl6 mice fed a high fat diet for 12 weeks [44]. Most of the genes were up- or down-regulated depending on the fat depot; only 138 genes were commonly regulated in both sexes. For skeletal muscle, the most obvious sexual dimorphism is the greater muscle mass in men [46]. DOR expression might be controlled, at least in part, by sex hormones as estrogen receptor beta (ERb) knock-out (Glenmark et al., 2004) leads to increased DOR expression in skeletal muscle (GDS791). The dynamics of DOR expression control at the transcriptional level are currently being investigated.

## Conclusions

In summary, control of DOR expression is shown to exhibit complex regulation and to depend on dietary fat content, tissue type and sex. DOR might exert important effects on the function of certain tissues by gene regulation or via autophagy and counteract adipogenesis in high fat diets or obesity. Further investigation into differential DOR expression in tissues where its expression was shown to vary with respect to obesity is necessary to uncover DOR’s role in metabolic diseases.

## Competing interests

The authors declare that they have no competing interests.

## Authors' contributions

CF-D participated in RNA isolation as well as statistical analysis and wrote the manuscript. OL carried out RNA isolation and qPCR analysis in white as well as brown adipose tissue and wrote parts of the manuscript. SvdH and LO performed statistical analysis under the supervision of TB. NB carried out RNA isolation and qPCR analysis in muscle tissue. SH, JB and FK participated in expression analysis in adipose and muscle tissue. UR housed, bred and sacrificed mice. AH, BB and TB co-developed the strategy and edited the manuscript. BGB conceived the project, supervised experiments, coordinated the project and edited the manuscript. All authors read and approved the final manuscript.

## Supplementary Material

Additional file 1 **Primers used in the study for qPCR.** Sequences of primers used in this study for qPCR with their annealing temperatures (T°an) and amplification efficiencies. Click here for file

Additional file 2 **DOR expression in mice fed a normal (ND) or fat rich diet (FD/HFD).** 40 days old NMRI mice kept at standard conditions were administered either a fat diet (FD; 18% fat) or a high fat diet (HFD; 80% fat) for 1 week. Control group animals received a normal fat diet (ND; 3.3% fat) during all time of the experiment. DOR expression in fat (WAT, BAT) and muscle (SM, HM) tissues was quantified by qPCR. The expression data were normalized with housekeeping genes and calibrated to reference mice. Mean values and standard deviation (sd) per tissue type, gender and diet group are shown. “n” indicates the number of animals in each group. Click here for file

Additional file 3 **Changes in DOR expression in mice fed a fat rich diet (FD/HFD).** 40 days old NMRI mice kept at standard conditions were administered either a fat diet (FD; 18% fat) or a high fat diet (HFD; 80% fat) for 1 week. Control group animals received a normal fat diet (ND; 3.3% fat) during all time of the experiment. DOR expression in fat and muscle tissues was quantified by qPCR. All the expression data from qPCR were normalized with housekeeping genes. Normalized data of FD and HFD mice were compared to those of ND animals using Welch Two Sample t-tests for independent samples. “up/down“ indicates differences in DOR expression of FD and HFD mice in comparison to control animals with respective p-values. “-“ indicates that differences were not significant. “n“ indicates the number of animals in each group. Click here for file

Additional file 4 **Influence of tissue type on DOR expression in male mice fed a fat rich diet (FD, 18% fat content).** Influence of muscle tissue type (HM and SM) on DOR expression was proved by ANOVA. We found significant influence of tissue type in male mice in FD animals (normalized to control animals). DOR expression was higher in HM than in SM. Click here for file

Additional file 5 **DOR expression in fat and muscle tissues of genetically obese mice.** DOR expression was quantified by qPCR in fat and muscle tissues of genetically obese (DU6/DU6i) and normal (DUKs/ DUKsi) mice of both sexes at the age of 45 or 100 days post natum (p.n.). All the expression data from qPCR were normalized with housekeeping genes. Normalized data of DU6/DU6i were compared to those of DUKs/ DUKsi mice using Welch Two Sample t-tests for independent samples. “up/down“ indicates differences in DOR expression of DU6/DU6i mice in comparison to control animals with respective p-values. “-“ indicates that differences were not significant. “n“ indicates the number of animals in each group. Click here for file

Additional file 6 **DOR expression in genetically obese (DU6/DU6i) and normal (DUKs/DUKsi) mice.** DOR expression was quantified by qPCR in fat (WAT, BAT) and muscle (SM, HM) tissues of genetically obese (DU6/DU6i) and normal (DUKs/DUKsi) mice at the age of 45 or 100 *days post natum* (*p.n*.). The expression data were normalized with housekeeping genes and calibrated to reference mice. Mean values and standard deviation (sd) per tissue type, gender, age and genetic group are shown. “n” indicates the number of animals in each group. Click here for file

Additional file 7 **Influence of tissue type on DOR expression in 45 days old male DU6i mice.** Influence of adipose tissue type (BAT and WAT) on DOR expression was proved by ANOVA. We found significant influence of tissue type in 45 days old male DU6i mice (normalized to DUKsi mice). DOR expression was higher in WAT than in BAT. Click here for file
