# Supplementary material for: Extrinsic and intrinsic regulation of DOR/TP53INP2 expression in mice: effects of dietary fat content, tissue type and sex in adipose and muscle tissues
Source: Nutr Metab (Lond). 2012 Sep 21;9:86. doi: 10.1186/1743-7075-9-86 (PMC3497704; doi:10.1186/1743-7075-9-86)
Supplement: Additional file 1 — Primers used in the study for qPCR. Sequences of primers used in this study for qPCR with their annealing temperatures (T°an) and amplification efficiencies. [file 1743-7075-9-86-S1.pdf]

Additional file 1 - Primers used in the study for qPCR.

Sequences of primers used in this study for qPCR with their annealing temperatures (T°an) and amplification efficiencies.

gene	forward primer (5'→ 3' direction)	reverse primer (5'→ 3' direction)	T°an	efficiency %
**ACTb** (Beta-actin, cytoplasmic)	GGGAAATCGTGCGT GACATC	GCGGCAGTGGCCA TCTC	58°C	99.5
**B2M** (Beta-2 microglobulin)	GCCTGTATGCTATC CAGAA	GAAAGACCAGTCCT TGCTGA	58°C	96.0
**DOR (TP53INP2)** (transformation related protein 53 inducible nuclear protein 2)	AACCACAGCCTGCT TCTAATACCTT	TCAGCCAGTCTCAA CACAAAACAC	58°C	96.1
**GUSb** (Beta-glucuronidase)	AATGAGCCTTCCTC TGCTCT	AACTGGCTATTCAG CTGTGG	58°C	97.6
**PPIA** (Peptidylprolyl isomerase A)	AGCTCTGAGCACTG GAGAGA	GCCAGGACCTGTAT GCTTTA	58°C	104.1
