# Supplementary material for: Extrinsic and intrinsic regulation of DOR/TP53INP2 expression in mice: effects of dietary fat content, tissue type and sex in adipose and muscle tissues
Source: Nutr Metab (Lond). 2012 Sep 21;9:86. doi: 10.1186/1743-7075-9-86 (PMC3497704; doi:10.1186/1743-7075-9-86)
Supplement: Additional file 2 — DOR expression in mice fed a normal (ND) or fat rich diet (FD/HFD). 40 days old NMRI mice kept at standard conditions were administered either a fat diet (FD; 18% fat) or a high fat diet (HFD; 80% fat) for 1 week. Control group animals received a normal fat diet (ND; 3.3% fat) during all time of the experiment. DOR expression in fat (WAT, BAT) and muscle (SM, HM) tissues was quantified by qPCR. The expression data were normalized with housekeeping genes and calibrated to reference mice. Mean values and standard deviation (sd) per tissue type, gender and diet group are shown. “n” indicates the number of animals in each group. [file 1743-7075-9-86-S2.pdf]

Additional file 2 - *DOR* expression in mice fed a normal (ND) or fat rich diet (FD/HFD).

40 days old NMRI mice kept at standard conditions were administered either a fat diet (FD; 18% fat) or a high fat diet (HFD; 80% fat) for 1 week. Control group animals received a normal fat diet (ND; 3.3% fat) during all time of the experiment.

DOR expression in fat (WAT, BAT) and muscle (SM, HM) tissues was quantified by qPCR. The expression data were normalized with housekeeping genes and calibrated to reference mice. Mean values and standard deviation (sd) per tissue type, gender and diet group are shown. “n” indicates the number of animals in each group.

tissue	diet	sex	mean values	sd	n
**white adipose tissue (WAT)**	FD	male	1.39	+/- 0.34	7
		female	0.83	+/- 0.19	6
	HFD	male	1.09	+/- 0.23	6
		female	0.29	+/- 0.18	6
	ND	male	0.80	+/- 0.33	6
		female	0.97	+/- 0.37	6
**brown adipose tissue (BAT)**	FD	male	2.43	+/- 0.35	7
		female	2.86	+/- 0.65	6
	HFD	male	2.09	+/- 0.58	6
		female	3.33	+/- 1.53	6
	ND	male	2.51	+/- 0.51	6
		female	2.44	+/- 0.69	6
**skeletal muscle (SM)**	FD	male	0.61	+/- 0.23	12
		female	0.47	+/- 0.23	10
	HFD	male	1.20	+/- 0.58	6
		female	0.92	+/- 0.43	6
	ND	male	0.75	+/- 0.38	6
		female	0.57	+/- 0.30	6
**heart muscle (HM)**	FD	male	0.67	+/- 0.40	11
		female	1.11	+/- 0.78	11
	HFD	male	1.05	+/- 0.54	6
		female	0.83	+/- 0.72	6
	ND	male	0.40	+/- 0.24	6
		female	0.38	+/- 0.25	6
