# Supplementary material for: Extrinsic and intrinsic regulation of DOR/TP53INP2 expression in mice: effects of dietary fat content, tissue type and sex in adipose and muscle tissues
Source: Nutr Metab (Lond). 2012 Sep 21;9:86. doi: 10.1186/1743-7075-9-86 (PMC3497704; doi:10.1186/1743-7075-9-86)
Supplement: Additional file 3 — Changes in DOR expression in mice fed a fat rich diet (FD/HFD). 40 days old NMRI mice kept at standard conditions were administered either a fat diet (FD; 18% fat) or a high fat diet (HFD; 80% fat) for 1 week. Control group animals received a normal fat diet (ND; 3.3% fat) during all time of the experiment. DOR expression in fat and muscle tissues was quantified by qPCR. All the expression data from qPCR were normalized with housekeeping genes. Normalized data of FD and HFD mice were compared to those of ND animals using Welch Two Sample t-tests for independent samples. “up/down“ indicates differences in DOR expression of FD and HFD mice in comparison to control animals with respective p-values. “-“ indicates that differences were not significant. “n“ indicates the number of animals in each group. [file 1743-7075-9-86-S3.pdf]

Additional file 3 - Changes in *DOR* expression in mice fed a fat rich diet (FD/HFD).

40 days old NMRI mice kept at standard conditions were administered either a fat diet (FD; 18% fat) or a high fat diet (HFD; 80% fat) for 1 week. Control group animals received a normal fat diet (ND; 3.3% fat) during all time of the experiment. *DOR* expression in fat and muscle tissues was quantified by qPCR. All the expression data from qPCR were normalized with housekeeping genes. Normalized data of FD and HFD mice were compared to those of ND animals using Welch Two Sample t-tests for independent samples. “up/down” indicates differences in *DOR* expression of FD and HFD mice in comparison to control animals with respective p-values. “-” indicates that differences were not significant. “n” indicates the number of animals in each group.

tissue	diet	sex	p-value (up/down)	n
**white adipose tissue (WAT)**	FD	male	0.0138 (up)	7
		female	0.5972 (-)	6
	HFD	male	0.1324 (-)	6
		female	0.001926 (down)	6
**brown adipose tissue (BAT)**	FD	male	0.8365 (-)	7
		female	0.3171 (-)	6
	HFD	male	0.2411 (-)	6
		female	0.4017 (-)	6
**skeletal muscle (SM)**	FD	male	0.5845 (-)	12
		female	0.3768 (-)	10
	HFD	male	0.09653 (-)	6
		female	0.1368(-)	6
**heart muscle (HM)**	FD	male	0.2164 (-)	11
		female	0.02009 (up)	11
	HFD	male	0.01602 (up)	6
		female	0.381 (-)	6
