# Supplementary figures and images for: Extrinsic and intrinsic regulation of DOR/TP53INP2 expression in mice: effects of dietary fat content, tissue type and sex in adipose and muscle tissues
Source: Nutr Metab (Lond). 2012 Sep 21;9:86. doi: 10.1186/1743-7075-9-86 (PMC3497704; doi:10.1186/1743-7075-9-86)

Tissue influence on DOR expression  
**Fat diet (18%) male mice,  $p < 0.05$**

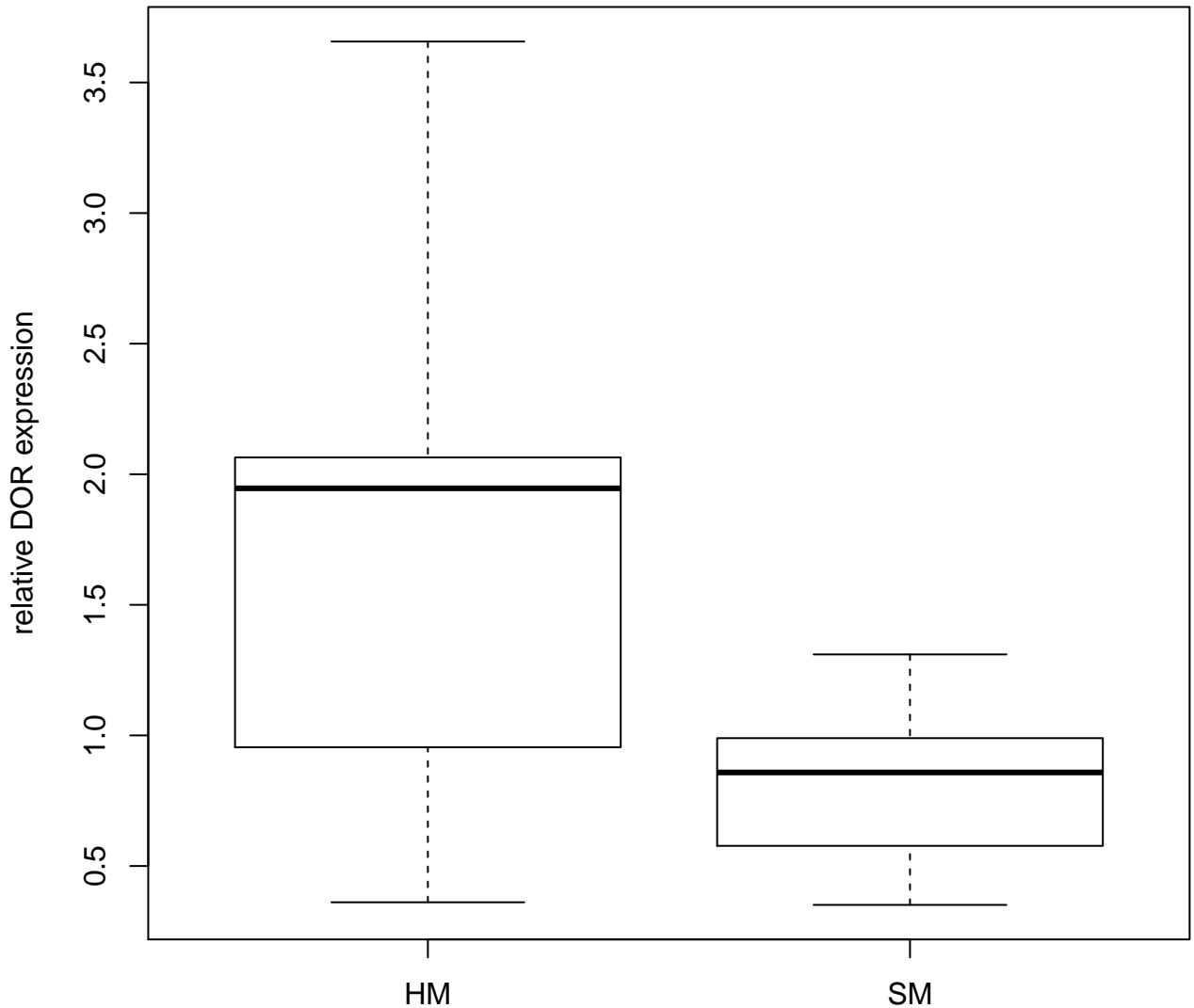

Supplement: Additional file 4 — Influence of tissue type on DOR expression in male mice fed a fat rich diet (FD, 18% fat content). Influence of muscle tissue type (HM and SM) on DOR expression was proved by ANOVA. We found significant influence of tissue type in male mice in FD animals (normalized to control animals). DOR expression was higher in HM than in SM. [file 1743-7075-9-86-S4.pdf]

Tissue influence on DOR expression  
**45 days old male mice,  $p < 0.01$**

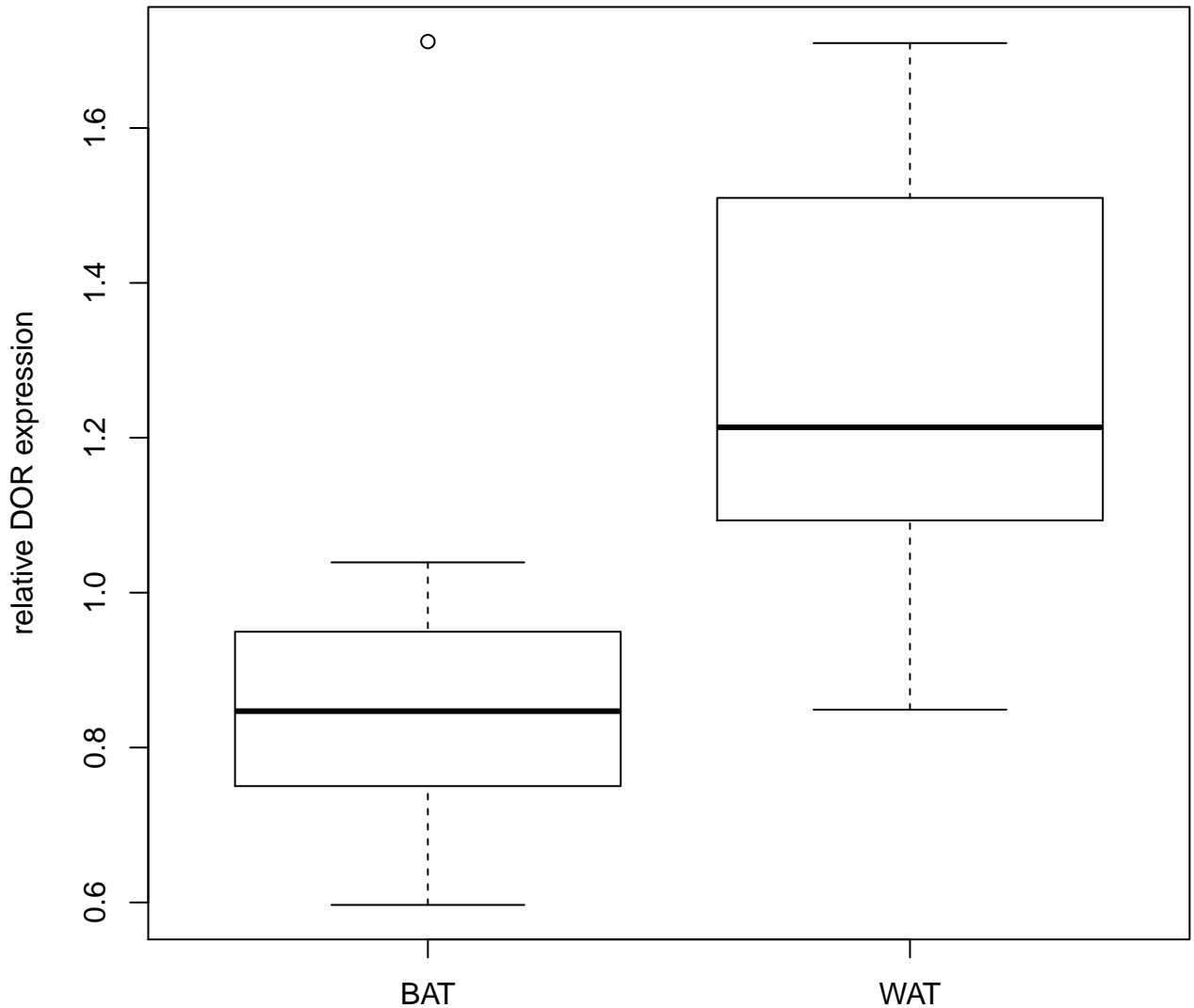

Supplement: Additional file 7 — Influence of tissue type on DOR expression in 45 days old male DU6i mice. Influence of adipose tissue type (BAT and WAT) on DOR expression was proved by ANOVA. We found significant influence of tissue type in 45 days old male DU6i mice (normalized to DUKsi mice). DOR expression was higher in WAT than in BAT. [file 1743-7075-9-86-S7.pdf]
