# Supplementary material for: Extrinsic and intrinsic regulation of DOR/TP53INP2 expression in mice: effects of dietary fat content, tissue type and sex in adipose and muscle tissues
Source: Nutr Metab (Lond). 2012 Sep 21;9:86. doi: 10.1186/1743-7075-9-86 (PMC3497704; doi:10.1186/1743-7075-9-86)
Supplement: Additional file 5 — DOR expression in fat and muscle tissues of genetically obese mice. DOR expression was quantified by qPCR in fat and muscle tissues of genetically obese (DU6/DU6i) and normal (DUKs/ DUKsi) mice of both sexes at the age of 45 or 100 days post natum (p.n.). All the expression data from qPCR were normalized with housekeeping genes. Normalized data of DU6/DU6i were compared to those of DUKs/ DUKsi mice using Welch Two Sample t-tests for independent samples. “up/down“ indicates differences in DOR expression of DU6/DU6i mice in comparison to control animals with respective p-values. “-“ indicates that differences were not significant. “n“ indicates the number of animals in each group. [file 1743-7075-9-86-S5.pdf]

Additional file 5 - *DOR* expression in fat and muscle tissues of genetically obese mice. *DOR* expression was quantified by qPCR in fat and muscle tissues of genetically obese (DU6/DU6i) and normal (DUKs/DUKsi) mice of both sexes at the age of 45 or 100 days *post natum* (*p.n.).* All the expression data from qPCR were normalized with housekeeping genes. Normalized data of DU6/DU6i were compared to those of DUKs/DUKsi mice using Welch Two Sample t-tests for independent samples. “up/down” indicates differences in *DOR* expression of DU6/DU6i mice in comparison to control animals with respective p-values. “-” indicates that differences were not significant. “n” indicates the number of animals in each group.

**tissue**	**age days *p.n.***	**mouse strains**	**sex**	**p-value (up/down)**	**n**
**white adipose tissue (WAT)**	45	DU6i	male	0.06815 (-)	12
		DU6	female	0.1478 (-)	10
	100	DU6	male	0.4699 (-)	5
		DU6	female	0.06361 (-)	5
**brown adipose tissue (BAT)**	45	DU6i	male	0.3987 (-)	10
		DU6	female	0.2317 (-)	8
	100	DU6	male	0.6844 (-)	5
		DU6	female	0.01531 (up)	5
**skeletal muscle (SM)**	45	DU6i	male	0.5517 (-)	12
		DU6	female	0.9541 (-)	10
	100	DU6	male	0.4895 (-)	5
		DU6	female	0.08204 (-)	5
**heart muscle (HM)**	45	DU6i	male	0.01678 (down)	12
		DU6	female	0.6918 (-)	10
	100	DU6	male	0.02344 (up)	5
		DU6	female	0.1294 (-)	5
