# Supplementary material for: Extrinsic and intrinsic regulation of DOR/TP53INP2 expression in mice: effects of dietary fat content, tissue type and sex in adipose and muscle tissues
Source: Nutr Metab (Lond). 2012 Sep 21;9:86. doi: 10.1186/1743-7075-9-86 (PMC3497704; doi:10.1186/1743-7075-9-86)
Supplement: Additional file 6 — DOR expression in genetically obese (DU6/DU6i) and normal (DUKs/DUKsi) mice. DOR expression was quantified by qPCR in fat (WAT, BAT) and muscle (SM, HM) tissues of genetically obese (DU6/DU6i) and normal (DUKs/DUKsi) mice at the age of 45 or 100 days post natum (p.n.). The expression data were normalized with housekeeping genes and calibrated to reference mice. Mean values and standard deviation (sd) per tissue type, gender, age and genetic group are shown. “n” indicates the number of animals in each group. [file 1743-7075-9-86-S6.pdf]

Additional file 6 - *DOR* expression in genetically obese (DU6/DU6i) and normal (DUKs/DUKsi) mice.

DOR expression was quantified by qPCR in fat (WAT, BAT) and muscle (SM, HM) tissues of genetically obese (DU6/DU6i) and normal (DUKs/DUKsi) mice at the age of 45 or 100 days *post natum* (*p.n.*). The expression data were normalized with housekeeping genes and calibrated to reference mice. Mean values and standard deviation (sd) per tissue type, gender, age and genetic group are shown. “n” indicates the number of animals in each group.

**tissue**	**age days *p.n.***	**mouse strains**	**sex**	**mean values**	**sd**	**n**
**white adipose tissue (WAT)**	45	DU6i	male	1.49	+/- 0.31	12
		DU6	female	2.26	+/- 0.74	10
	45	DUKsi	male	1.18	+/- 0.39	12
		DUKs	female	2.65	+/- 0.43	10
	100	DU6	male	1.54	+/- 0.53	5
		DU6	female	0.99	+/- 0.27	5
	100	DUKs	male	1.30	+/- 0.16	4
		DUKs	female	0.73	+/- 0.11	5
**brown adipose tissue (BAT)**	45	DU6i	male	1.23	+/- 0.41	10
		DU6	female	1.31	+/- 0.24	8
	45	DUKsi	male	1.34	+/- 0.30	10
		DUKs	female	1.46	+/- 0.22	8
	100	DU6	male	2.73	+/- 0.85	5
		DU6	female	3.14	+/- 0.23	5
	100	DUKs	male	2.61	+/- 1.25	5
		DUKs	female	2.22	+/- 0.56	5
**skeletal muscle (SM)**	45	DU6i	male	1.21	+/- 0.53	12
		DU6	female	0.44	+/- 0.11	10
	45	DUKsi	male	1.10	+/- 0.25	12
		DUKs	female	0.45	+/- 0.15	10
	100	DU6	male	3.29	+/- 2.27	5
		DU6	female	2.43	+/- 0.96	5
	100	DUKs	male	3.51	+/- 0.80	5
		DUKs	female	3.92	+/- 1.31	5
**heart muscle (HM)**	45	DU6i	male	0.19	+/- 0.03	12
		DU6	female	0.32	+/- 0.12	10
	45	DUKsi	male	0.22	+/- 0.03	12
		DUKs	female	0.32	+/- 0.06	10
	100	DU6	male	1.90	+/- 0.66	5
		DU6	female	0.66	+/- 0.12	5
	100	DUKs	male	0.98	+/- 0.70	5
		DUKs	female	1.54	+/- 1.18	5
